# Too many tags spoil the metadata: investigating the knowledge management of scientific research with semantic web technologies

**DOI:** 10.1186/s13321-019-0345-8

**Published:** 2019-03-21

**Authors:** Samantha Kanza, Nicholas Gibbins, Jeremy G. Frey

**Affiliations:** University of Southampton, Southampton, SO17 1BJ UK

**Keywords:** Semantic tagging, Ontologies, Scientific documents, Document management

## Abstract

Scientific research is increasingly characterised by the volume of documents and data that it produces, from experimental plans and raw data to reports and papers. Researchers frequently struggle to manage and curate these materials, both individually and collectively. Previous studies of Electronic Lab Notebooks (ELNs) in academia and industry have identified semantic web technologies as a means for organising scientific documents to improve current workflows and knowledge management practices. In this paper, we present a qualitative, user-centred study of researcher requirements and practices, based on a series of discipline-specific focus groups. We developed a prototype semantic ELN to serve as a discussion aid for these focus groups, and to help us explore the technical readiness of a range of semantic web technologies. While these technologies showed potential, existing tools for semantic annotation were not well-received by our focus groups, and need to be refined before they can be used to enhance current researcher practices. In addition, the seemingly simple notion of “tagging and searching” documents appears anything but; the researchers in our focus groups had extremely personal requirements for how they organise their work, so the successful incorporation of semantic web technologies into their practices must permit a significant degree of customisation and personalisation.

**Electronic supplementary material:**

The online version of this article (10.1186/s13321-019-0345-8) contains supplementary material, which is available to authorized users.

## Introduction

The development and evolution of the Web has sparked a new age of “unprecedented communication and data exchange between scientists” [1]; and technologies centered around the web have continued to grow and evolve with techniques such as semantic web technologies being used to provide context and meaning to data [2]. We have progressed into a digital age where using electronic devices is the norm and a vast breadth of information is now stored and made available digitally. In keeping with this progression, Electronic Lab Notebooks (ELNs) were created as replacement for the paper lab notebook to store scientific research in a digital format. However, despite the many reasons to store ones research digitally, and the considerable uptake of using ELNs (or at least some form of software platform) in industry, the uptake of ELNs in academia remains very low [3].

However, just because academic institutions are not using ELNs en mass, it does not mean that they are not producing digital copies of their scientific research. While earlier studies of scientific laboratory practice demonstrated that scientists typically preferred to write up their lab work on paper [4, 5], later work by Monteiro et al. [6] demonstrated a shift in these practices whereby more digital resources are being created by scientists. Studies performed by Kanza et al. [7] concluded that typically a large proportion of scientists’ formal documents are written up electronically, and that Ph.D. students (the study participants referred to in this paper) build up a significant quantity of electronic documents over the course of their degrees. Furthermore, although many studies show that scientists remain unconvinced about tools such as Electronic Lab Notebooks (ELNs) [8–10], they are mindful of the ways in which software and new technologies can improve their current workflow practices, such as semantic web technologies [6, 11–13].

When producing scientific reports, a significant amount of different documents are often used to provide the necessary information; ranging from experiment plans and data to observation notes and literature notes. It is vitally important to consider how to manage this vast level of scientific research data in an efficient manner. Utilising semantic web technologies provides the ability to use open standards to expose research data as formalised metadata [13], and to link the different sets of data collected throughout the experimental process [12]. These technologies facilitate interoperability and enable documents to be tagged and categorised to improve organisation and search capabilities [13]. In this regard, when compared to manual paper-based methods, searching can be performed both faster and more accurately with the aid of technology, and a semantic search can be very powerful across a large corpus of documents as it facilitates a search based on meanings and concepts as opposed to just text [14, 15].

**Fig. 1:**
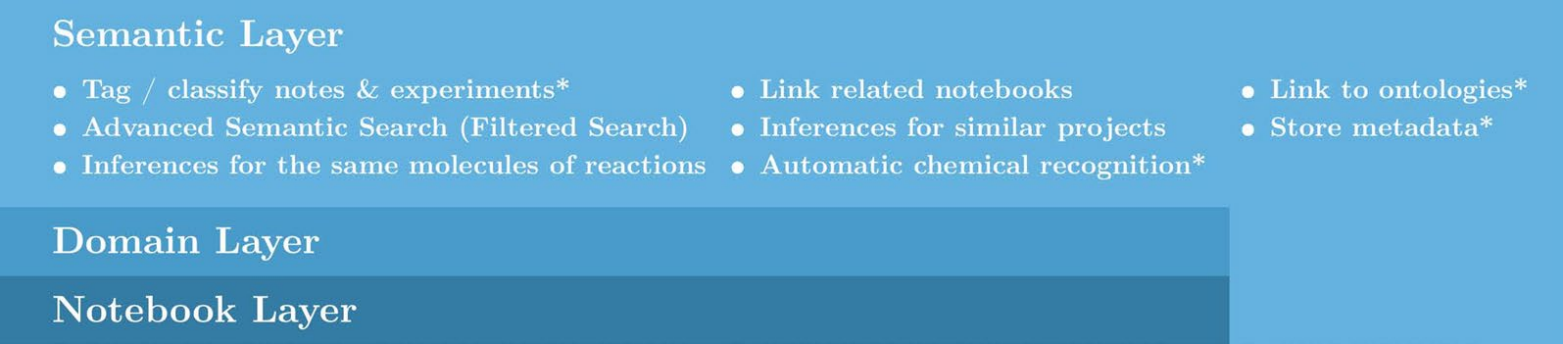
A summary of the user-desired features of an ELN, adapted from Kanza et al. [7] to illustrate the desired semantic features

User studies performed by Kanza et al. [7] examined a number of factors relating to ELNs, ranging from querying scientists about adoption barriers, current ELN usage, asking a set of user-specific questions before and after trialling an ELN, and conducting focus groups to study current laboratory practice. The combined results of these studies produced a three-tiered model of the user desired features of a Semantic ELN with the features broken down into generic notebooking features, domain-based features and semantic features, as demonstrated in Fig. 1, which outlines a simplified version of the model presented in Kanza et al. [7].

The scientific uses cases for each of the semantic features detailed in Fig. 1 are given below. The use cases are presented using a simplified template adapted from the use cases templates detailed by Fox and McGuinness [16], and for each use case the primary actor will be the ELN user(s).

**Table:** 

*Use Case Name:* Tag/classify notes and experiments	
*Goal:* Semantically tag and assign categories to scientists notes and experiments.	
*Summary:* If experiments and notes are tagged and assigned classifications then users can search for documents relating to a particular subject (e.g. a specific experiment or certain types of experiments).	
*Preconditions:* User loads documents into ELN, link to ontologies, automatically detect chemicals.

**Table:** 

*Use Case Name:* Link Related Notebooks	
*Goal:* Suggest links between related notebooks in a research group.	
*Summary:* If research groups collaborate and share their notebooks, suggestions can be made between related notebooks to highlight potential new collaborations and show users similar work to theirs.	
*Preconditions:* Multiple Notebooks are created and shared, tag/classify notes and experiments.

**Table:** 

*Use Case Name:* Link to Ontologies	
*Goal:* Link terms in notes to concepts and relationships in standard vocabularies (ontologies).	
*Summary:* By aligning terms with common vocabularies, documents can be classified and tagged in a consistent manner, and a semantic search can be implemented.	
*Preconditions:* Identify relevant ontologies.

**Table:** 

*Use Case Name:* Advanced Semantic Search	
*Goal:* Search by concepts and relationships rather than text.	
*Summary:* Searching by concepts and relationships is substantially more powerful than searching by text. Users can search for specific chemicals, or experiments performing certain actions.	
*Preconditions:* User loads documents into ELN, tag/classify notes and experiments.

**Table:** 

*Use Case Name:* Inferences for Similar Projects	
*Goal:* Infer similar projects that could be linked to a users project.	
*Summary:* If research groups collaborate and share their notebooks, inferences can be made about similar projects being conducted by different researchers to highlight potential collaborations and facilitate knowledge sharing.	
*Preconditions:* Multiple Notebooks are created and shared, tag/classify notes and experiments.

**Table:** 

*Use Case Name:* Store Metadata	
*Goal:* Automatically store metadata about notes/experiments.	
*Summary: *Storing metadata about notes and experiments enhances indexing and searching capabilities.	
*Preconditions:* User loads their documents into the ELN, tag/classify notes and experiments.

**Table:** 

*Use Case Name:* Inferences for the same molecules of reactions	
*Goal:* Make inferences to suggest which users are working on similar molecules of reactions.	
*Summary:* If research groups collaborate and share their notebooks, inferences can be made to enable users to find out who is working on similar molecules of reactions.	
*Preconditions:* Built in understanding of molecules.

**Table:** 

*Use Case Name:* Automatic Chemical Recognition	
*Goal:* Automatically detect chemicals that are reference in notes/experiments.	
*Summary:* Automatically detecting chemicals in documents means that users can search through experiments that use certain chemicals.	
*Preconditions:* User loads documents into ELN, link to ontologies.	

This paper follows on from the work conducted in Kanza et al [7] by looking to address some key research questions pertaining to the use of semantic web technologies in ELNs, to ascertain how this semantic layer could work in practice and establish the knowledge management requirements of scientists, and indeed whether putting knowledge management provisions in place would make a difference to their work processes. This paper looks to address the following research questions:

1. What are the potential advantages of using semantic web technologies to manage scientific research?
2. What tools and technologies are the most effective for using semantic web technologies to manage scientific research?
3. What are the needs and requirements of scientists with regards to knowledge mangement of their research?
4. Would the use of semantic web technologies convince scientists to digitise their research further?

These research questions were investigated in a qualitative manner, in a bid to gain further understanding of how these technologies could be successfully implemented in ELNs and Scientific Knowledge Management Tools. A detailed methodology of the research is given, demonstrating how the different tasks have been broken down to answer the research questions; followed by a description of each task. These tasks are then detailed, including the initial literature and technical investigations, followed by a description of the work undertaken to create a prototype (Semanti-Cat) which was created partially to investigate what tools and technologies were most suited to be used in this body of work, and also as an evaluation tool to understand the use cases for these technologies, and to elicit knowledge management requirements from scientists. The results of the study are then presented and discussed with reference to the different research questions. The paper then summarises the main conclusions and key findings and outlines the potential for future research.

## Study methodology and design

This study was designed as a follow on to the work conducted in Kanza et al. [7]. The different pieces of research undertaken for that paper were collated together and used to propose a three layered Semantic ELN Platform. However, whilst some work has been conducted to evaluate the use of semantic web technologies in an ELN platform, and some tools have been produced to provide semantic resources that could be used in a Semantic ELN (e.g. ontologies and tagging systems), there are several research gaps to address; namely how to implement these technologies effectively, and what scientists actually want from a knowledge management perspective.

**Fig. 2:**
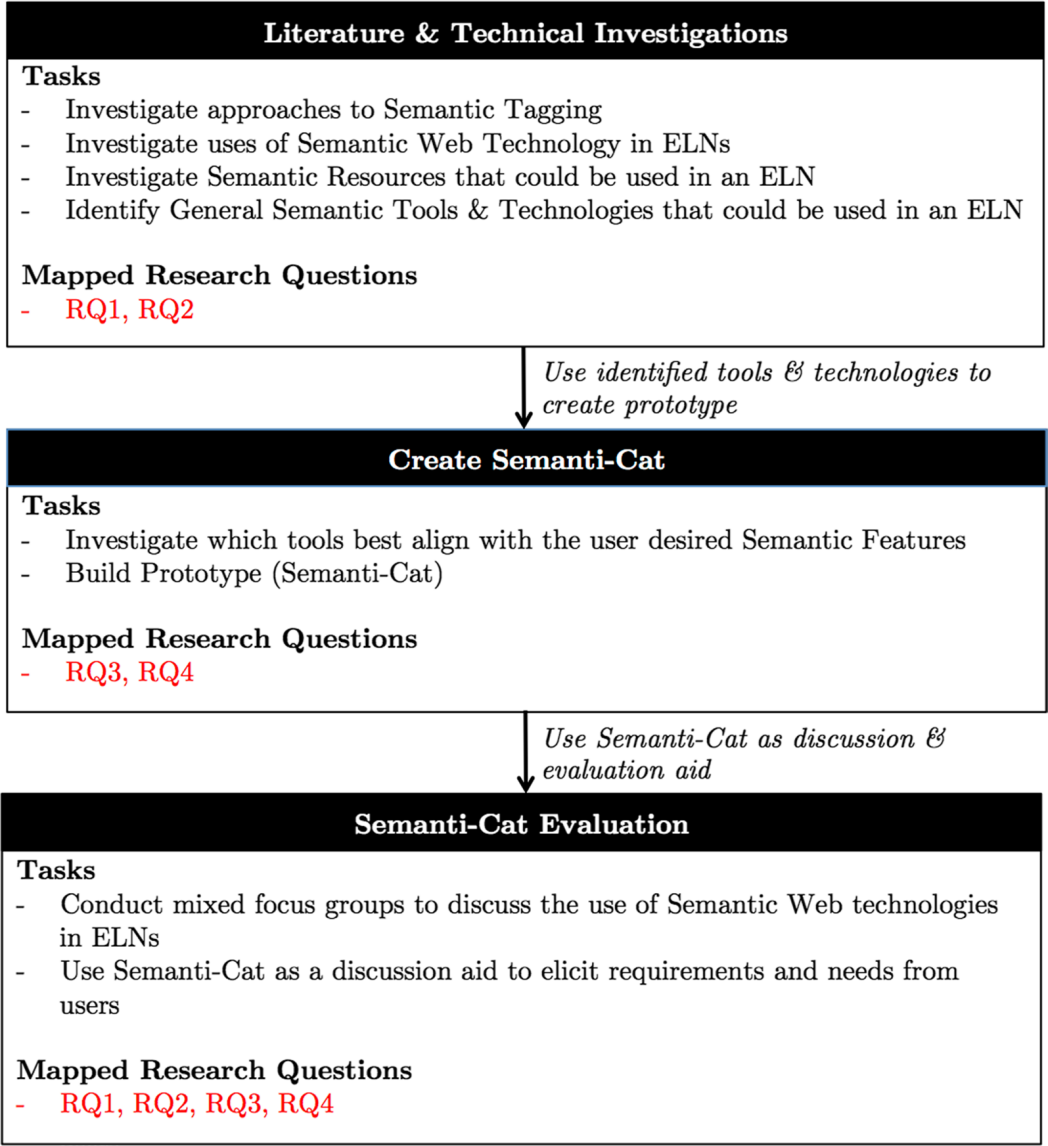
Methodology diagram of the overall study conducted by this paper

In order to answer these questions, Fig. 2 details the different components of the study.

The literature and technical investigations were used to identify the current state of semantic web technologies in this domain, both with respect to where these technologies had already been used in ELN projects and also to understand the availability of semantic resources that could be used in a semantic ELN. From these investigations, these tools were then reviewed to see which could be put together in a prototype. Semanti-Cat was then created as a discussion and evaluation aid to allow the participants of the focus groups a way to understand the types of technologies that could be used, to see which tools and ontologies worked best for tagging and to provide them with a mechanism to channel more structured feedback and to initiate discussions not only about the specific pieces of functionality, but also regarding the overarching elements of knowledge management.

## Literature and technical investiations

The idea of using semantic web technologies for knowledge management is not a new one; this is a vast research space and there are many different aspects of research and different types of approaches to be considered when undertaking studies in this area.

### Semantic tagging approaches

Knowledge management is a complex field, and requires different approaches depending on the nature of the information that requires managing, and the users involved with a given system. With respect to creating metadata for linking related objects and facilitating advanced searches there are a number of different approaches.

One approach is user generated metadata. It is becoming more common across certain social tagging systems (e.g. Flickr) that have a wide spread of users and a particular type of information to manage, to set up lightweight structures called folksonomies; whereby the users themselves create their metadata by assigning specific tags and categories to their items. This is advantageous in that the users do the bulk of the work and categories and tags can be generated quite quickly with a low technical effort. The downside of this approach is that it lacks consistency and organisation, it makes it harder for users to search through, and runs the risk of discrepancy (e.g. reading could refer to an activity or the city) [17]. Specia et al. [18] undertook a project to address these issues by explicitly defining the semantics behind this tagging space by cleaning the tags, analysing tags for co-occurence, clustering related tags and finally linking them to semantic concepts in ontologies. This meant that users could search across concepts or relationships rather than by text, vastly improving the accuracy of their results. Similarly Hotho et al. [19] created an algorithm based on PageRank to rank different folksonomy tags during searching to provide a more accurate way of searching through the user generated metadata.

The opposite approach is to generate the metadata automatically with a tagging service. There are some generic tagging services available: Thomson Reuters OpenCalais [20] and Ontotext’s Semantic Annotation [21] are two similar tools. Both offer the ability to automatically categorise and tag documents using algorithms that make use of Wikipedia’s data and tags to suggest the most relevant tags from those categories to be assigned to a document.

There are also text processing tools available which can be used to annotate documents to derive tags from them. GATE [22] is a generic open source text processing and analysis tool which tokenises documents into separate numbers, punctuation and different parts of English speech. It comes with a range of plugins—the most specific to this study being one called ChemistryTagger which was built to extract and tag chemical compounds and chemical elements. Similarly Hawizy et al. [23] created ChemicalTagger, an NLP (Natural Language Processing) tool to annotate chemistry experiment documents by tokenising the text and using a combination of OSCAR4 [24], domain-specific regular expressions, and English taggers to break the document down into chemical elements, compounds, and different parts of English speech. Berlanga et al. [14] used a statistical framework to annotate, index, and search life science documents; however, this was created for use on a corpus of documents and, rather than using a human component, was evaluated against other services to see how their annotations compared. Similarly, Piao et al. [25] created a historical thesaurus semantic tagger that assigned user-generated thematic categories to a document corpus, which was evaluated by comparing manually annotated documents to semantically annotated documents.

There are advantages and disadvantages to these approaches, the latter has the capacity to produce more consistent tags and categories, such that searching across these descriptions will be more accurate. However, this is assuming that the automatically generated tags are accurate and representative of what the user wanted to store about their information. Equally, whilst the user generated approach ensures that the user can accurately describe their resource, the lack of consistency from user generated tags makes searching substantially more difficult. An ideal bridge between the two would be to automatically generate most of the tags for the user, but permit them to make changes and refine the tags. As evidenced by the studies undertaken by White [26] and Kanza et al. [7], scientists organisation and management of their data is “completely individual and unique”, and so these unique needs must be taken into account, whilst also being wary of ensuring consistency across managed information.

### Scientific ontologies

Work has also been conducted to create scientific ontologies, which could serve as useful resources in a semantic ELN even if this was not the original development intention. Ontologies are dictionaries or vocabularies for the semantic web [27] that provide the formal definitions of common terms (as classes) used in a specific domain, and the hierarchy and relationships between those classes. The research towards this paper identified several ontologies in the different scientific domains.

The Royal Society of Chemistry developed three ontologies: The Named Reactions Ontology RXNO [28] which contains formal descriptions of connected organic name reactions to their roles in an organic synthesis; the Chemical Methods Ontology CMO [29] which describes methods, instruments and some material artefacts used in chemical experiments, and the Molecular Processes Ontology MOP [28] which describes chemical processes that take place at the molecular level, e.g methylations and electron transfer. In addition to this, there is also the Chemical Information Ontology CHEMINF [30] which includes terms for the descriptors commonly used in cheminformatics software applications and the algorithms which generate them.

A number of ontologies for the biological domain have also been created. The Plant Ontology PO [31] links together plant related terms, the Cell Ontology CO [32] contains a structured vocabulary for animal cell types, and the Gene Ontology GO [33] defines concepts and classes used to describe gene functions, and the relationships between these concepts. There were limited offerings for physics ontologies, although one exists to cover the classes and properties typically used by astronomers, which is the Astrophysics Ontology PHYSO [34].

There are also some ontologies that span multiple scientific domains. The Chemical Entities of Biological Interest ChEBI Ontolgoy [35, 36] is a dictionary of molecular entities focused on ‘small’ chemical compounds, and the NanoParticle Ontology NPO [37] represents the basic knowledge of physical, chemical and functional characteristics of nanotechnology as used in cancer diagnosis and therapy.

These ontologies cover a range of the terms that could appear in a scientists ELN. They are not an exhaustive list, but would serve as a useful starting point.

### Semantic web technologies in ELNs

Talbott et al. [12] used an ELN client developed by the Pacific Northwest National Laboratory to investigate the use of semantic web technologies within an ELN [38]. This ELN focused on storing its data in underlying repositories, exposing its metadata in RDF and integrating with other systems to produce and consume metadata. Whilst this ELN might have been discontinued, the work done concluded that taking the data from ELNs and exposing it as standard metadata (which other ELNs such as Labtrove have also been focusing on [39]) is an important step in bringing the ELN into the wider world of the “semantic web and knowledge grids”. In addition, they felt that creating ELNs that are capable of producing a shared record with underlying semantics will be a “key enabler of next generation research”.

There has also been some work done in the area of Semantic Notebooks as a general concept. Drăgan et al. [40] focuses on some of the affordances of the semantic web; namely how to interlink important information and how to design interfaces to support the existing workflow of the user. This work facilitated a study of users to see whether they preferred SemNotes (a new Semantic Notebook) or Evernote, which produced favourable results with respect to SemNotes. This demonstrates that these additional features can even outperform a product as popular as Evernote. This survey was conducted before Evernote started incorporating Semantic Web technologies within their context booster,^1^ that provided links to additional content on the web to enrich users notes.

### Conclusions

However, despite these attempts, there is no semantic chemistry notebook available in the market. Additionally, most of this work has been disjointed from actual electronic lab notebook software, such as creating ontologies or a semantic middleware platform that could be used alongside an ELN. This means that despite the affordances of semantic web technologies, and despite researchers consistently recognising the part they could play in an ELN, they have yet to become part of the recognised package of an ELN. This is an area that has been investigated in great detail on an academic level [9, 11, 13, 41] and clearly has some worthwhile affordances as described in this section. Furthermore, whilst work has also been carried out in applying semantic tagging and annotations to scientific documents; typically these have typically taken the form of creating new semantic tagging methods or new methods of searching semantic tags, and evaluating them against specific criteria rather than evaluating the ideals of semantic tagging and subsequently searching from a user’s perspective, particularly within a specific domain; which is an area this paper looks to address.

## Semanti-Cat implementation

**Fig. 3:**
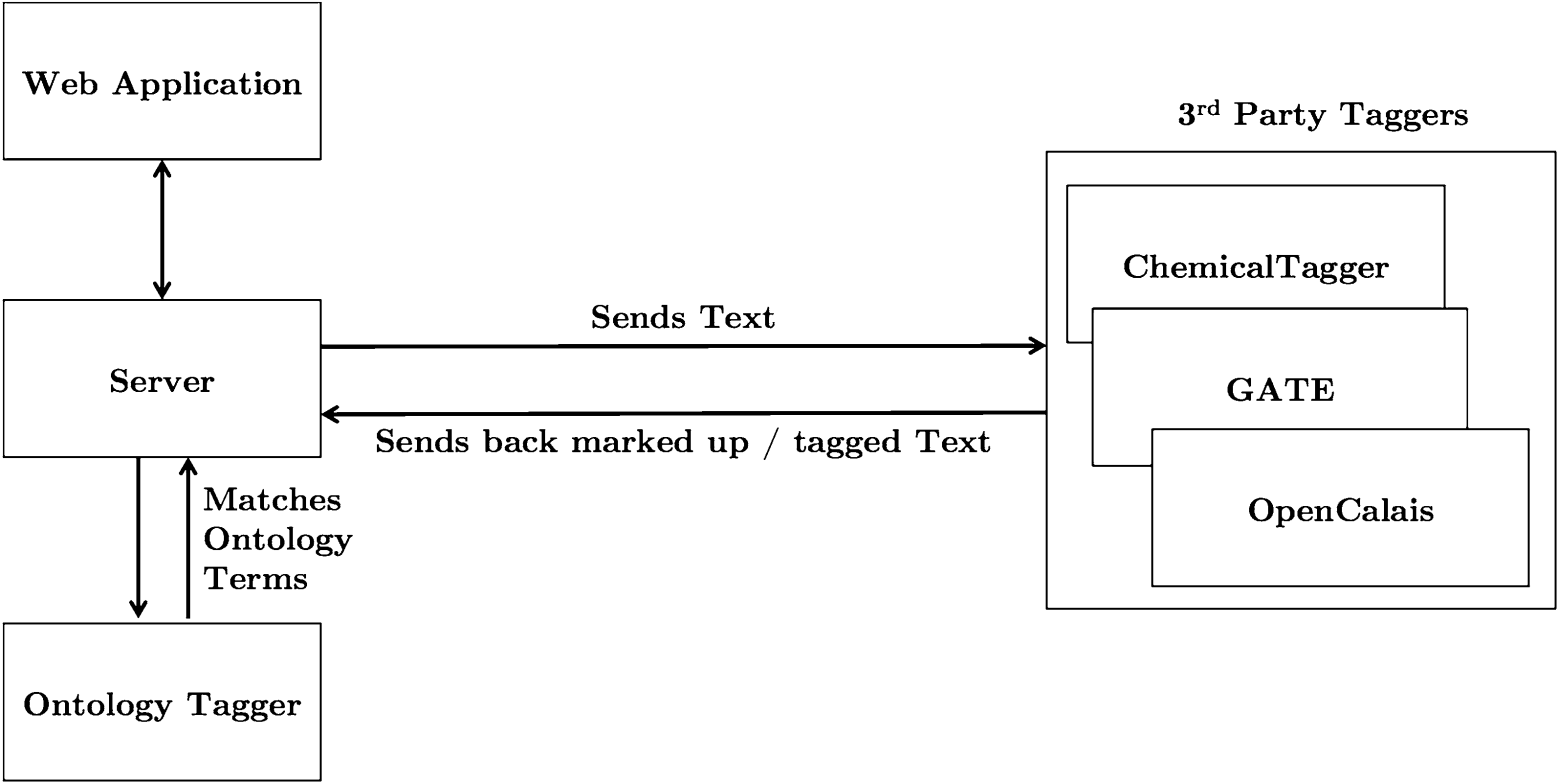
Architecture of Semanti-Cat

This section details the overall implementation of Semanti-Cat [42], detailing the features that were implemented and the subsequent choices that were made regarding which of the identified tools and technologies to use. The implemented features of Semanti-Cat concentrated on the starred features in Fig. 1 (tagging and classifying notes and experiments, linking to ontologies, automatic chemical recognition services and storing metadata), the reason for which is that (1) these features can be trialled on single documents rather than requiring a large corpus (which would be needed for features such as inferences for the same molecules or projects or to link related notebooks); and (2) as evidenced by the use cases in the “Introduction”,   before implementing links or advanced search techniques, the appropriate tagging methods need to have been applied. For the purposes of the prototype the metadata that will be stored is just the tags and the chemicals extracted for each document. A basic search was then implemented to give the participants a focus to discuss how they would want a semantic search to work for them. After identifying the tools for this project, the first prototype was created as a Java Web Application. Java enabled use of the Apache Jena [43], an open source java framework for creating semantic web applications. The high-level architecture of the system is detailed below in Fig. 3.

As Fig. 3 illustrates, for the purposes of the initial prototype, the web application then runs documents through the OpenCalais API and the two chemical recognition services, ChemicalTagger and GATE. Finally, these documents were also matched against the chosen ontologies (detailed below) to see what terms from there existed in the document. The sections below describe the how the different tools and technologies were selected and implemented in more detail.

### Tag/classify notes and experiments

Two classification services were identified in the initial investigations: Thomson Reuters OpenCalais [20] and Ontotext’s Semantic Annotation [21]. Both offered similar features and utilised Wikipedia’s data and tags in their algorithms to produce document tags. However, OpenCalais both provided the option of “social tags” for document classification (whereas OntoText had yet to release this feature) and more detailed documentation, and therefore was selected as the primary tagging service. The tags for each document were extracted from OpenCalais and stored as metadata. The OpenCalais API was accessed using an HTTPClient Post to send a request containing the documents to tag, and the API sends back a HTTPResponse with the document marked up with social tags that can be extracted. Below is a list of the request headers detailing the different parameter options and types that were defined in the OpenCalais User Guide [44], and explaining which value was chosen for each header.

- *X-AG-Access-Token:* License key for the API.
- *Content-Type:* mimeType of the document(s) being sent. Options: text/html (web pages), text/xml (xml documents), text/raw (clean, unformatted text), and application/pdf (PDF files as binary streams, only available to premium users). For this first implementation, ‘*text/raw*’ was chosen.^2^
- *omitOutputtingOriginalText:* Boolean parameter with options of true (send back original document content in response) or false (omit this content). This was set to ‘*true*’ as the original document content was not needed.
- *x-calais-contentClass:* Genre of the input document. Options: none (default value), news (news stories) or research (for research papers, but only supported for PDF files). For this first implementation ‘*none*’ was chosen.^3^
- *x-calais-language:* Document Language. Options: English, French or Spanish. This was set to ‘*English*’.
- *x-calais-selectiveTags:* Metadata tags to be used to classify the documents: There were several options for this parameter but the most relevant was ‘*socialtags*’, which attempt to classify the document as a whole, based on the Wikipedia folksonomy.
- *outputFormat:* Response output format to the HTTPClientPost. Options: xml/rdf (default), application/json and text/n3. The default value of ‘*xml/rdf*’was chosen as any of these could have been parsed to extract the tags.

### Automatic chemical recognition

The main usable chemical recognition services identified in the initial investigations were GATE [22] with the use of the ChemistryTagger plugin, and ChemicalTagger [45]. Chemical Tagger is a natural language processing tool that aims to identify and markup chemicals in text. GATE is a more general natural language processing tool but it does have a ChemTagger extension so this was also used alongside ChemicalTagger in the hope that between them most of the chemicals featured in the users text would be automatically identified. The identified chemicals for each document are also stored as metadata for the document alongside the social tags.

GATE can be used in two main ways for text processing, firstly as a standalone application [46], and secondly within another program (GATE Embedded). Java provides two ways to use GATE Embedded for marking up documents, the GATE environment can be fully constructed from scratch in Java, or a version of the GATE Application that denotes the appropriate setup and plugins to use can be loaded in and constructed [39] (for simplicity in this instance, the second option was chosen). GATE returns the documents with partial XML-style markup around the identified chemicals which were then extracted using simple regex matching.

ChemicalTagger was simple to implement, as the jar files can be downloaded from the ChemicalTagger website and run with a few lines of code. Initial code was written to create a default instance of the ChemistyTagger which calls a tokeniser, the oscarTagger (which tags chemicals), the domain specific regexTagger that looks for the formation of chemistry actions, and the openNLPTagger which uses natural language processing to tag different English phrases such as nouns and verbs. These different taggers were then run over the scientific documents, followed by running a sentence parser to split up the document into sentences. Finally, the output XML Document was created containing the original document text split up into XML tags. This document was then parsed using a standard SAX parser to strip out the OSCAR4 chemical tags and action.

### Link to ontologies

In order to use ontologies in this prototype, the Semantic Library Jena [43] was used. Unfortunately, Jena only facilitates loading ontologies into the system in their entirety, meaning that some of the ontologies identified in the “Literature and technical investiations” section were too large to be loaded in. For Chemistry the Named Reactions Ontology (RXNO), Chemical Methods Ontology (CMO) and Molecular Processes Ontology (MOP) were all used. For biology, the only two that were small enough to be read into the system were PO—Plant Ontology [31] and CL—Cell Ontology [32]. For Physics, the sole ontology that was identified (the AstroPhysics Ontology) was also able to be read into the system. These ontologies were taken from the ones identified in the “Scientific ontologies” section.

The ontology matching code was written for this project, and looked for matches of terms within the different ontology classes and these were also added as tags in the first instance. Where the ontology contained descriptions of the terms these were also stored so that they could be displayed as hover text for matched ontology terms. In the first iteration of this project all the identified tags, chemicals and ontology terms were kept and stored as metadata in relation to the documents. The logic behind this was to show the participants in the focus groups a wide range of potential tags and chemicals to see which ones they actually would use.

**Fig. 4:**
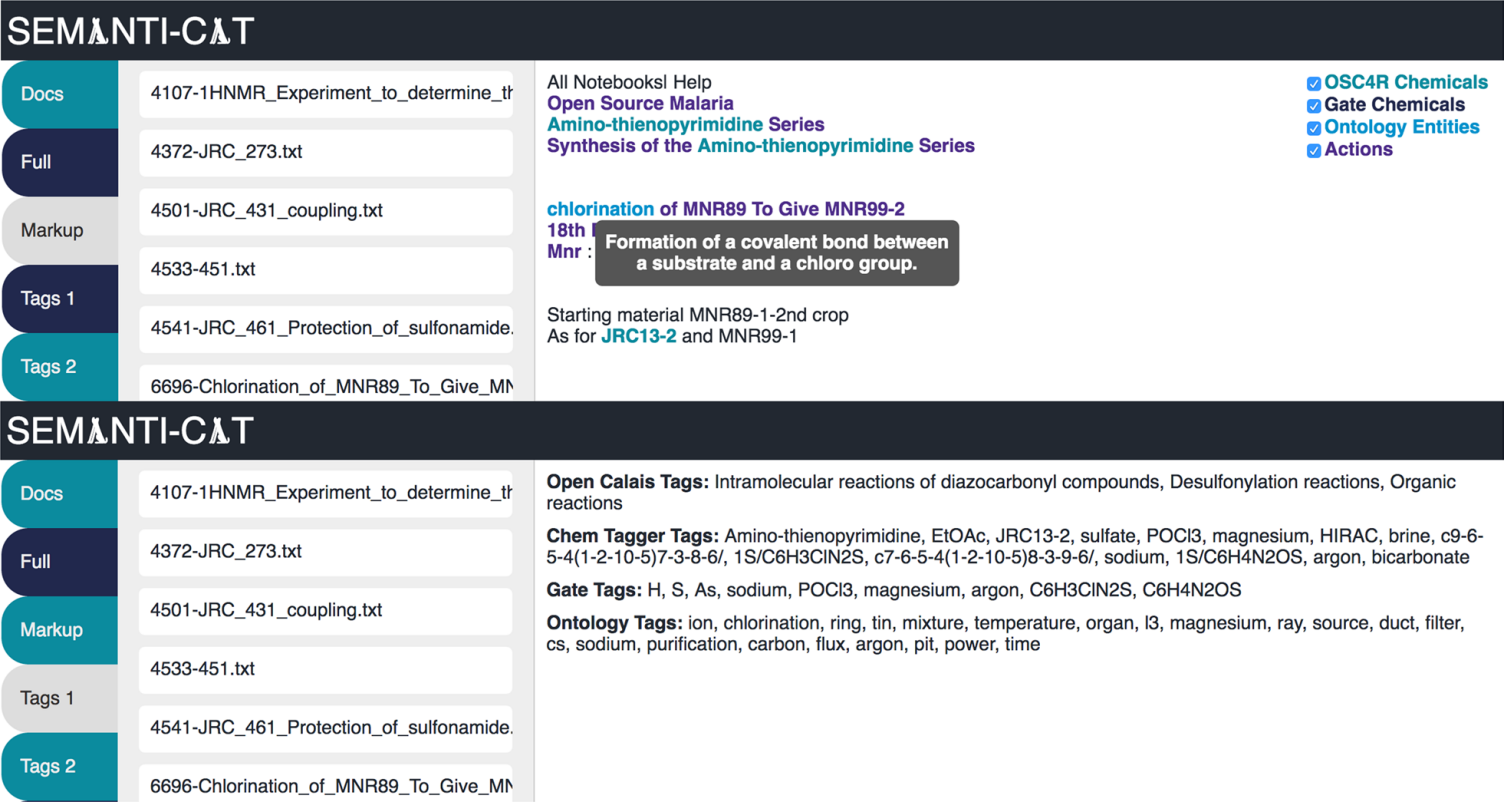
Semanti-Cat—markup tab (shows highlighted tags and tooltips) and Tags 1 tab (shows tags/chemicals from different services)

For the initital setup of Semanti-Cat, each ontology was loaded in and the classes and annotations were iterated over to produce a set of classes with their descriptive labels (which would later be used as the tooltip text for the ontology tags - as demonstrated in Fig. 4). It runs a simple algorithm over each document to match the ontology terms within the documents, iterating over each ontology term to see if it existed within the document, and if so assigning it as a tag and producing the tooltip markup such that the front-end web interface of Semanti-Cat would have the text to use in the tooltip. This was done in a very basic way for this first iteration to test how many ontology terms would be matched and to see which tags the participants of the focus groups thought were useful.

### Basic search

The initial searching technologies used in this first prototype are quite basic with the aim to improve them based on user feedback about both how they wanted the search to work and how they wanted their documents to be tagged (with what weightings) and marked up in the first place. The basic search was initially implemented using pure term frequency [47]. For each search term *t*, a term frequency score is computed based on the weight of that term, which is the number of occurrences in the document *d*, which equates to *tf*_*t*,*d*_. The same operation is then applied to the title *T* *tf*_*t*,*T*_. These two are added together for each term in the search box to give an overall term frequency score for each document, and then this score orders the results. Two slightly advanced search options are also offered: *text:searchTerm* and *title:searchTerm* should the user wish to restrict the searching to just the text or title. Part of the focus group questions asks the participants how they would search for different documents and where they would expect certain terms to have priority, such as in the title or in the text.

### User interface

Semanti-Cat was designed as a simple web application to illustrate to the focus group members which tags had been assigned by which service to their documents. The user interface has the following tabs:

- Docs: This tab shows the document in its original text form so the user can see the text they submitted.
- Full: This tab shows both the tags and the markup (explained below).
- Markup: This tab shows the document marked-up with the different types of tags (and the actions from ChemicalTagger). Each tagging service has a colour so that the user can see which tag came from which service. The markup from each service can be turned off to aid the users in seeing how each service tagged their documents and also to identify overlap where certain terms have been tagged by more than one service. The mark-up also offers tooltips. Figure 4 shows the tooltip that appears when the user hovers over the word chlorination, with the text of the tooltip coming from the descriptive label assigned to the class chlorination in the Molecular Processes Ontology. These descriptions are to provide further information about the tags where possible.
- Tags 1: This tab displays lists of the different tags arranged by tagging service (OpenCalais, ChemicalTagger, GATE and the Ontology Tags). This allows participants to see a quick view of all of the different tags assigned to their documents such that they can understand how each one has currently been categorised and comment on how well this has been done.
- Tags 2: This tab is very similar as it arranges it the tags into two groups: OpenCalais tags, and the rest of the tags. This is because the OpenCalais tags are terms that will not necessarily be in the document itself as they categorise the document, whereas GATE and ChemicalTagger recognise chemicals that exist in the documents and use them as tags; similarly the ontology tags are also formed of terms that exist in the document.

These tabs are meant to provide the participants of the focus groups a quick and easy way of understanding how their documents have been tagged. Figure 4 shows screenshots of the Markup and Tags 1 tab.

## Evaluation

The evaluation focus groups comprised fifteen Postgraduate students from the University of Southampton, studying Physics, Chemistry or Biology. The anonymised participant information is listed below; links to the ethics application and transcriptions of these focus groups are all listed in the Additional file 1.

- Chemistry Focus Group: 6 Participants L, J, S, AP, AJ and AK
- Physics Focus Group: 4 Participants A, B, AL and AM
- Biology Focus Group: 5 Participants Q, R, AN, AO and AQ

Semanti-Cat was evaluated through semi-structured focus groups, with questions to encourage discussion around certain topics, but which were open-ended enough to leave the forum of discussion open for participants to debate and talk around the subjects raised [5]. Semanti-Cat was deliberately designed in a generic way to allow the participants to discuss the way the documents have been tagged and marked up, to facilitate discussions of what they want from these pieces of functionality and to elicit both their requirements and identify potential use cases from the participant feedback.

The focus groups begin by attempting to understand what types of documents a scientific semantic tagging system would be expected to process. Participants were asked to send a document each to the study coordinator to be marked up prior to the study to demonstrate the range and variation of documents. The participants’ first discussion point was to describe the document they had sent and detail how typical it was of their usual work, and then to comment on the standard makeup of the documents they work with (e.g. how much of their documents were made up of words/diagrams/pictures), and finally what formats they typically create their documents in (e.g. .txt, .pdf, .doc). This was to build up a picture of not only the types of documents a tagging system would have to work with, but also the format of them, as images and diagrams are by nature much more complex to tag or assign information to, and formats such as PDFs are harder to extract data from than plain text documents.

Following this, the different tagging systems were discussed and compared. The participants were each shown which tags had been assigned by which service to their documents and asked to identify which were the most appropriate and inappropriate (if any) for their work and if they felt that there were any obviously missing tags. This was both to understand how well the tagging systems worked, and also whether different ones worked better for documents from different scientific disciplines. The participants were also asked how they would personally tag their documents and whether they would make use of the options to add and remove their own tags. This was to get a better idea of how different participants would expect their documents to be tagged and how much involvement they would want to have with that process.

The topic of search was next, which aimed to explore how the participants expected a search to work, and what types of advanced search options (if any) they desired. One of the aims of these focus groups was to understand how to create an advanced semantic search to meet the user requirements detailed in the studies performed by Kanza et al. [7], with the look to refine the tagging process based on user feedback and then design a search feature that would fit with the users’ needs.

After search, the markup was discussed. Semanti-Cat had marked up the documents to show which terms and actions were pulled out by which tagging service, both to illustrate the difference between the third party services but also to show how much of the document information was extracted into tags. Additionally, descriptions of the types of tags were put in tooltips that could be viewed by hovering over the tags. The ontology tags detailed the descriptions given in the ontology as part of their tooltip to provide the user with further information, the chemical elements that were identified had tooltips that identified them as chemical elements and the different actions pulled out by ChemicalTagger were also marked up and given tooltips that detailed what type of action they were. The participants were asked how (if at all) they would want to see their documents marked up, and whether they thought the tooltips were useful.

## Results

This section details the results of the focus groups, covering first the different types of documents that the participants brought, and then their comments on the tagging, searching and markup elements of Semanti-Cat. Prior to taking part in the focus group, the participants were given the deliberately limited guidance of “You will be asked to send a scientific document (either one of your own or one that you feel bears similarity to the kind of work you do)” to give them the freedom to send over a document that they would want to be semantically tagged. The results from both previously recorded studies in the literature [4] and the initial user studies in Kanza et al. [7] illustrated that the notes that scientists produced were very personal and differed greatly; therefore it was important to fully trial this system with a range of documents to ensure that the contrasting needs of different scientists can be met. The range of documents sent over varied significantly (as shown in Table 1).

**Table 1:** Comparison of the document types, lengths, and how many of each type of tag was applied to it by Semanti-Cat

Participant/document information	Discipline	Document type	Document format	Document makeup	# Pages	# Words	# Chars	# Open Calais tags	# Ontology tags	# ChemicalTagger tags	# GATE tags
Participant L	Chemistry	Thesis section	.DOC	Mostly text, 1 figure	4	990	5120	12	17	16	4
Participant J	Chemistry	Blog post	HTML	Mostly structure diagrams with some text	2	441	1919	6	2	15	0
Participant S	Chemistry	ESI document	.DOC	~ 60% text ~ 40% figures	12	2216	14,108	12	75	92	3
Participant AI	Chemistry	Thesis section	.DOC	Mostly text, 1 figure	4	1501	9562	12	61	62	4
Participant AJ	Chemistry	Handwritten lab book page	Handwritten	Mostly text and some filenames	1	160	1229	3	5	10	0
Participant AK	Chemistry	Experimental writeup	.DOC	Roughly equal text/figures	14	1558	8453	7	32	26	5
Participant A	Physics	Research paper	.PDF	~ 60% text ~ 40% figures/equations	8	5996	35,398	12	79	92	2
Participant B	Physics	Sample report	.DOC	Roughly equal text/figures	4	711	4396	8	13	8	0
Participant AL	Physics	Handwritten lab book page	Handwritten	Little text and sketched diagram	1	75	723	0	1	3	0
Participant AM	Physics	Handwritten lab book page	Handwritten	100% text	1	173	1070	8	4	0	0
Participant Q	Biology	Thesis section	.DOC	Mostly words, some figures/graphs	1	378	2478	10	18	8	0
Participant R	Biology	Thesis section	.DOC	Mostly words, some figures/graphs	3	899	5754	12	25	12	0
Participant AN	Biology	Experiment writeup	.DOC	Mostly words, some figures/graphs	12	3160	21,276	7	34	60	0
Participant AO	Biology	Literature report	.DOC	Mostly words, some figures/graphs	10	3449	23,780	12	38	58	1
Participant AQ	Biology	Experiment writeup	.DOC	Mostly words, some figures/graphs	7	2492	13,861	12	19	20	1

Table 1 details the different types of documents sent over by each participant. The Document Type denotes what type of document the extract came from, and the Document Format shows what format the document was sent over in. For the purposes of this simple prototype the documents were converted into plain text to be run through Semanti-Cat. This was fairly straightforward with the .DOC offerings, but the PDF proved more challenging. A PDF parser was used which managed to strip out most of the words, but the results were not as smooth as if the document had been in a .DOC form. The handwritten documents obviously required typing up before they could be run through the system. The Document Makeup shows whether the document consists of text, figures or both. This was to see how common the use of diagrams were and to understand the different makeup of the documents that would be put into an ELN. As is demonstrated in the pages, words and chars columns, pages did not necessarily correlate to words (e.g. Participant AK who had a 14 page document, which had a roughly equal range of text and figures, but the figures were quite large and therefore took up a lot more space.

These metrics already illustrate not only the range of pieces of work that Ph.D. students produce but also the different content that a semantic tagging platform would need to handle, including content that is both challenging to tag and mark up or even digitise in the first place in some instances (figures, sketches or diagrams), and content that users may not want tagged, such as reference lists. Further to this, asking the participants about what formats they typically created their documents in and what their general mark-up was with regards to words and pictures and diagrams elicited a similar diversity. The biologists were fairly uniform, stating that they predominantly produced documents that were mostly words with some figures and tables, and that their figure captions would be quite detailed, and all agreeing that they created their documents in Microsoft Word, and used software such as PRISM, GraphPad or Microsoft Excel to model their data and produce their graphs.

### Tagging and chemical recognition services

There was some variance among the different disciplines with regard to how the participants felt about the different tagging and chemical recognition services but there were also some common themes that came out of these discussions. Almost every participant regarded OpenCalais as too generic and broad. In each focus group one person said that of all four services OpenCalais was probably the most useful, but even they prefixed that statement with an opinion that they were still too broad. Participant J (a chemist) commented “if it was for other people looking into a much larger system than this then it could be more useful”, identifying that the generic descriptions the group had been given by OpenCalais of ‘chemistry’, ‘organic chemistry’ and ‘electrochemistry’ were not very useful when that was their scientific specialisation and in all likelihood all of their documents would be tagged as such, whereas in a larger system of varying disciplines or subdisciplines these tags would provide a more useful differentiation.

Similarly, when evaluating the ontology tags, most participants said that they found them too generic and also that too many tags were pulled out from them. For example, in Participant AP’s four-page document, Semanti-Cat pulled out 32 tags from different ontologies, which were deemed too many to helpfully classify a document. Additionally, in this document ‘voltammetry’ was identified as an ontology term, whereas OpenCalais identified ‘cyclic voltammetry’ as a tag, which Participant AP said, was more helpful. In Participant S’s 12-page document, 75 ontology tags were extracted; from which they highlighted six terms that they felt would be tags that they would use, that were either specific or highly related. Some participants thought that the ontology tags were useful in that they picked out the broad themes, although as Participant S stated: “I’m not going to search by theme, I know what I do and I want to know which experiment it is”.

There were some comments that not all of the ontology tags were relevant. Six different ontologies were used in Semanti-Cat (3 Chemistry, 2 Biology and 1 Physics), and the nature of the hierarchical structure of the ontologies means that some of the top level terms will be very generic words like ‘ring’ and ‘graph’ for example from the physics ontology. Additionally, some of the more generic tags assigned to documents of one discipline were from ontologies not specific to that discipline, but there were some crossovers in scientific terms especially at the top level of the hierarchy, meaning these were identified by more than one ontology, such as ‘group’ in the reactions ontology. Furthermore, there are some ontology terms that have more than one meaning, for example ‘current’ is a term in the physics ontology, and mixture a word in the chemical methods ontology; these words also have other meanings whereby it would not merit them being highlighted as a tag. This illustrates that a further level of natural language processing is necessary to both narrow down appropriate terms and rule out ones that are meant in a different context. It could make for a better tagging system if the OpenCalais tags were used to narrow down which ontologies should be considered and then write some extra natural language processing methods to tag only tag only certain terms.

With regards to chemical recognition tools, ChemicalTagger and GATE, the participants were unanimous in that they didn’t find GATE very useful. For several participants GATE did not identify any chemicals and for others it picked up only a few of the very common ones. GATE also often picks up chemicals wrongly as it suggests that C and H are chemicals because these can be chemical symbols, but also picks them up when they are not being used in that context. ChemicalTagger, as evidenced by Table 1, identified many more chemicals than GATE. Participant S was impressed that ChemicalTagger had picked out the IUPAC codes of the three compounds that they had made, commenting that “it’s more interesting to see what I’ve made” as opposed to just seeing the common structures. Some participants in each group said that they found ChemicalTagger had pulled out the best terms in relation to their work (3 Chemists, 2 Physicists and 4 Biologists). However, with similar comments to those regarding chemical recognition, ChemicalTagger was also described as too generic in places, in that it picked out all common chemicals.

The results of the initial user studies elicited a requirement for automatic chemical recognition, but listing them all as tags raised concerns. In addition to stating that too many generic chemicals being pulled out was not that useful, concerns were also raised about the accuracy of the chemical recognition, as some parts of equations were picked up as chemicals, and that it does not always accurately differentiate between chemical elements and chemical compounds, and is not consistent with identifying chemicals written in different ways that refer to the same structures (e.g. NH was written as HN in a chemical structure and these two were picked up individually). The participants that raised the most concerns regarding ChemicalTagger were the chemists, as they have made comments such as “this chemical would be used in everything I do” (for example, alumina for the electrochemists as they use that material to polish their electrodes). These comments have shown how, despite wanting chemicals to be automatically recognised, it does not mean that they should be used as tags, and that some Ph.D. students value picking out more individual elements of their work rather than picking out generic terms. Equally there were comments that the participants would not necessarily want to dispense with those chemical associations in certain situations, Participant J commented “I wouldn’t get rid of them, if you found a contamination of something in the lab and you had to look up every time you used it”, suggesting that there would be value in keeping them as metadata but without flagging them up as main descriptions of the document.

This was highlighted in the answers given when the participants were asked how they would want to tag their documents if they were doing it themselves. The chemists raised a wider spectrum of requirements for different types of tags than the physicists and biologists, but some common types of tags were requested across the different disciplines. There were some chemistry-specific tags that were asked for, and the rest of the tags fell into one of two categories: scientific tags such as experiment number or type, and more generic tags such as date or year. Table 2 illustrates the different tagging requirements that were elicited from the participants responses.

**Table 2:** Summary of tagging requirements elicited from the participants, organised by discipline

Category	Tag	Chemistry	Physics	Biology
Domain specific	Own compounds	✓		
	Molecules	✓		
	Sample number		✓	
Scientific	Experiment number	✓	✓	✓
	Experiment type	✓	✓	✓
	Experimental techniques/methods		✓	✓
	Measurements/units	✓		
	Key aims/conclusions	✓		
	Key results/findings	✓	✓	
Generic	Project name/number	✓	✓	
	Headings	✓		
	Date/year	✓		
	Broad themes			✓
	Filtered tags	✓		✓

Additionally, the participants also suggested that in addition to tagging their documents with different types of tags, they would like to know what types of documents they are, which would also enable them to search on different categories of documents. Another theme that emerged was that participants would expect a different level of tagging if they were tagging their work for themselves, or for other people, and that similarly they would expect to search through their own work differently to how they would approach searching through a colleagues work. This makes an interesting contrast to the results of the first focus groups, where some of the participants did not seem to consider putting processes in place for others to access their work either after they had left or if they were indisposed, and yet these participants were actively considering others making use of their work.

Furthermore, comments were made about how different levels of tags would be useful at different stages of ones academic career, in that a younger undergraduate might wish for more tags and be less concerned about picking out common things because they would just be starting their academic career, and additionally undergraduate work will vary more and have less of a very direct specialism than with a Ph.D. These themes illustrate that it is not just the tagging requirements that need to be taken into consideration here, it is also who the tagging is for. It is also clear that with the varying nature of what the Ph.D. students wished to be tagged, that there would need to be a high level of customisation of the types of tags that were assigned. This would also facilitate different levels of tagging for different groups. Hand in hand with these tagging considerations comes searching, as one of the motivations to associate these tags with documents is to create an improved semantic search that facilitates easy searching across users work.

### Searching

Following the tagging, the participants were asked how they would assume a search feature would work and what they would expect to be prioritised by the search; they were then asked about advanced search features. The current way the search works is detailed in the “Basic search” section, whereby a simple term frequency weighting is used for the main search across both the title and the main body of text, or the search can be restricted to consider only the title or the text body.

There was a disparity among the participants as to how they expected a search to work. Some participants said that they would expect the search to prioritise documents that had been tagged with the search phrase as a ‘highly weighted’ or ‘important’ tag. Others said that they would expect how often that term appeared in the document to be the first order of priority, and yet other participants said that they would expect to see the documents where the search term appeared in the title first. This simple question in itself illustrates how varying the search expectations and needs of different Ph.D. students can be, as even within the different disciplines there were contrasting opinions on this matter. It also illustrates how variable different scientists’ ways of working are, reaffirming that how scientists organise their work is a highly personal endeavour [4], and therefore how they would choose to search it is also equally personal. This links to the findings in Kanza et al. [7], which states that how scientists take notes and organise their work is a highly personal endeavour.

A theme that emerged from this set of questions was that most participants did wish for a more advanced search with additional options and restrictions. Several of these already appear in Google, such as using Boolean operators, searching on multiple terms and using regular expressions. Additionally, groups of participants agreed that they would want to be able to search by date (which was highlighted as a desired tag in the “Tagging and chemical recognition services” section), and that they would expect options to sort the searches by date and relevance, which are again typical searching features of Google and other search engines. There were also comments that bore some similar results to the previous focus groups detailed in Kanza et al. [7], which demonstrated that the chemists typically had more complex and varied methods of organising and searching through their data; and this was reflected in the responses given in these evaluations.

The main area that focused on a semantic search was being able to search on items that had been semantically tagged in the document and being able to filter the search by different types of tags, or drill down the hierarchy of tags. For example, searching for documents that had been tagged with experiment tags, and drilling down to the different experiment numbers. There were also desires for image searching, and the ability to search by InChIKey [48] or SMILES [49] structures to ascertain which documents contain these structures, which would also require having tagged the documents with these structures in the first place.

Similarly to tagging, the participants raised the point that the searching requirements and ways of searching would vary depending if one is searching through their own or another persons work. As Participant AK stated, “when it’s your own work you’re never searching broadly, you’re always searching specifically”, whereas the other participants pointed out that if they were searching through other people’s work or if other people were searching through theirs they would expect them to use a broader search with less refined options: “I know how to look through my stuff, but if push comes to shove I doubt I’d be able to find things in other people’s log books”—Participant B. There was also agreement that it would aid with knowledge transfer and encourage research groups towards better preservation of their data and work, if these enhanced services were designed well enough that they would actually be used. The participants comments showed that they would find this type of improved searching and tagging on other people’s work more useful rather than on their own work; as this would be work that they didn’t immediately know the order or context of. This illustrates that employing these software techniques can enable scientists to get more out of other researchers’ work.

These results suggest that both tagging and searching are very personal procedures for researchers, and that in order to design a search that would fit these contrasting needs, a lot more work would need to be done to ensure that both the tagging and searching was customisable. Additionally, further work would need to be done with the tagging to ensure that the right tags were captured, such that the scientists would be searching on the tags they require. The results also show that the Ph.D. students view how they use their work and how others use their work very differently, and that in some areas they see the benefits of this type of system being more for their peers or collaborators than for them directly.

### Markup and tooltips

The participants were then asked what they thought about the markup and tooltips, and whether they thought they were useful or not. With regards to the tooltips the similar theme of wanting to be able to customise the information re-emerged. Some of them liked the idea of having more information show up in the tooltips when hovering but wanted to be able to potentially customise that information as they felt it would be more useful to see it in their own words, and that there may be instances where their work was so specialised they would need to write the descriptions themselves. Similarly, there was a general feeling that for some of the more generic descriptions, if it was their own work they would know what the terms were that they had used and would not need a description: “If I’ve written centrifugation I know what it is”—Participant S. However, again it was pointed out that the more generic definitions given by the tooltips would potentially be very useful for other people reading their work, or younger students who hadn’t learned as much as they had. This bears similarities to the work done by Chen et al. [50] in the hypermedia world; their studies concluded that experts and novices exhibit different behaviour in using hypermedia learning systems, and require different levels of support. There was also resounding agreement that participants would want the options to turn the markup and tooltips on and off, and be able to customise information across multiple documents.

Some participants again made suggestions that it would be useful to break the tags down into types (see Table 1 for the different types of tags) so that they could turn different groups of tags on and off, and be able to search on different types of tags. It was also suggested that the tooltips could then link to other related work, Participant S suggested that “If it picked out my compound names and then does a tooltip that linked to all of the documents that had it in that it would be amazing” (which links back to the original user requirements FRS5 and FRS6 about making links between related notebooks and projects, which were not implemented as part of this first iteration). The responses suggested that some participants would make use of the markup and tooltips if they were both customisable and could be toggled on and off such that they didn’t have to be used or displayed at all time.

### Potential for digitisation improvement

The participants were asked whether they thought semantic tagging/improved search would have any impact on the efficiency of their work or how much they chose to digitise their work. Participant B stated that “this wouldn’t sway me to an ELN but would add to what we already have to make it easier to search through”, and Participant A said: “There is nothing you could do to an ELN for me that would make me use it on a day to day basis”, stating that they wouldn’t want to give up their lab notebook. Indeed, several times when asking the participants if they would consider using semantic web based software, the idea had to be decoupled from using an ELN and giving up their paper lab notebook just to elicit an answer.

These comments show how the very term ELN can come across as trying to replace the paper lab notebook rather than providing additional support to the lab process, suggesting that ELN based software might need to be marketed as a lab management tool or knowledge management tool rather than something that looks like it’s trying to be a direct electronic replacement. Participant S described the ELN that they tried as “a replacement for paper, and it’s taking something that works already and making it harder”. This also shows that there is still a resistance to ELN even if there are software requirements that participants have mentioned that they would like. Some participants said that the semantic tagging and searching would also encourage them to use an ELN but with the caveat that the tags and search features were actually what they wanted. Some of the physics participants stated that they would be more likely to use it on others work or their own work if they were giving it to others, rather than specifically on their own work for them. This suggests that ELNs might be better received if they weren’t marketed as a replacement to their paper lab notebook.

The participants provided mixed responses to whether they thought that the incorporation of semantic web technologies would improve their efficiency. Some of the chemists said that they thought it would make writing up easier if their documents were tagged and linked together, such that all the related material for an experiment could be easily searched for. However, the strong caveat was that this would only apply if the tags were deemed useful and met with what the participants wanted: “On these tags, no! On the tags I wanted, absolutely!”—Participant S. Some of the biologists thought that it would be useful, but it would require a level of personalisation to become fully useful: Such as personalising which terms to tag and anonymising the tag descriptions. Some of the physicists stated again that they thought the semantic tags would be more useful for other people’s work than their own. Again, this correlates with previous findings of the personalised nature of scientists organisation [7, 26]

The participants were then asked if they thought that semantic web technologies in an ELN (e.g. semantic tagging and searching) would encourage them to further digitise their work; this was also met with mixed responses. From the chemists, there was a desire expressed for semantic software to manage their work. Participant S stated that “it would need to be that all-encompassing project management tool, everything in one place”, and Participant J and AJ expressed a keenness for the tags, stating that they would find tagging and improved searching useful. Although the general feeling was that whilst the participants could get on board with tagging and adding their own custom tags, it wouldn’t necessarily encourage further digitisation, even if it would encourage using this type of software on their existing digitised work. A notable exception to this was Participant AK who said that they were looking to keep more stuff digitally and less stuff in a lab book as they felt that it was easier to store multiple copies, and mentioned their constant fear that the chemistry building might burn down and that all of their paper lab books would be destroyed.

The biologists stated that it wouldn’t necessarily encourage further digitisation, some of them took the stance that their work was already as digital as they felt they could get it, and one biology participant was quite against using solely technology for their lab write-ups, and there was an overall agreement that they all felt attached to their paper lab books, and were quite happy with the amount they digitised. From the physics focus group came a debate where some participants believed that students would need to be forced to start using ELNs from an early stage to ensure that they formed the habit of digitising their work, whereas other participants thought that if they had been forced to use one they would rebel against it. Participant Q of the biologists also stated that “if my supervisor had made me use one I would have gotten on with it...but even if I had an ELN I would still scribble on paper”. This shows a level of unwillingness to change formed habits and patterns (which fits comments from the initial user studies in [7] that once you’ve started something one way you do not want to change it); and clearly the benefits of paper and the hostile environment of the still heavily influences Ph.D. students decisions regarding how to handle their notes. Additionally, it is interesting that the participants who were most in favour of the use of an ELN and who either tried out ELN or generic note booking software were the youngest first year Ph.D. student, who potentially had come from a slightly younger generation that was more willing to use technology at an earlier stage, and the two Ph.D. students who had nearly finished and had secured jobs in industry and saw the benefits of using note booking software alongside their colleagues.

The final comments that were made also illustrated the perceived barriers to ELN software that are still very present. Paper is still viewed as easier to use than an electronic equivalent and some of the participants are firmly stuck in their habits of scribbling down on paper. Participant AL stated that “For me the biggest barrier would actually be digitising the work. I wouldn’t be prepared to...” And Participant Q stated that “Even if I don’t have paper I will get a bit of tissue and write on that and take it out with me instead of putting it in my phone or whatever because it’s easier”. Additionally, to strengthen this factor, there is still the conception that the software out there won’t be easy to use, Participant B stated “Strongly the issue isn’t the software, it is the hardware, writing stuff down or jotting stuff down is way easier on paper”. Participants still firmly believe that using paper in the lab is easier, and earlier comments highlighted that some perceive an ELN as a direct replacement that looks to replace paper but make things harder for them to do, which isn’t an attractive option. The participants do not believe that fully replacing their paper lab notebooks with an electronic version would make their life easier, so they are naturally against it.

## Discussion

Including participants from different scientific domains in this user study illustrated that scientists work in different ways, agreeing with the findings of Shankar [4] and Oleksik et al. [51]. The different groups of Ph.D. students showed some similar patterns to the focus groups conducted in Kanza et al. [7] with respect to discipline characteristics, reinforcing some of the different needs per discipline. The biologists were similarly uniform in their work both in terms of the documents they produced and the way they worked and even the content of their documents. They also all used a small set of software programs (Word, Excel, GraphPad and Prism) to produce their work and also mainly had more basic software needs that fitted with this proof of concept software. With respect to writing up and handling data they mainly used Word and Excel. Additionally, their documents were the easiest to process and tag as they were mostly words with some figures, and the figures had extensive captions, so all of the important information could be easily extracted from the documents. The physicists had some more technical needs, they primarily used LaTeX to produce their documents, meaning that they would have a high requirement for software like this to be able to handle PDFs; and had a high use of equations and figures (mostly with lesser captions than the biologists). They were more sceptical about the ability of the software to successfully extract and tag pieces of information from their documents, and also with regard to writing up their documents had a requirement for additional functionality such as handing equations. The chemists showed the greatest level of difference (similar again to the original focus groups in Kanza et al. [7]) and had contrasting opinions about what they would want tagged and produced more varying types of documents than the other two disciplines. This shows how contrasting the different science disciplines can be, and that chemistry in particular can vary greatly in terms of software needs and approach, and reinforces how personal an endeavour note taking is for the participants.

Given the degree of disparity between the disciplines, it is unsurprising that tagging and searching proved to be an equally personal endeavour for the participants, and that the simple-sounding notion of tagging scientific documents proved to be anything but simple. When the participants were asked what types of tags they would want, whilst some common themes were requested across the group, different participants prioritised different types of tags and ultimately each had a very personal idea about what they wanted. The participants desired a small amount of tags that related to the key distinguishing elements of their work, to enable them to effectively search through their documents. There were similar requests for selectivity with relation to automatically detecting chemicals in that participants stated that they did not see the value in having all the chemicals used identified as tags because some chemicals would be present in almost every document. For example, the electrochemists noted that ‘alumina’ would appear in all of their documents as they use it to polish their electrodes.

The participants demonstrated a degree of variance in their searching methods and expectations. For example, some participants use meaningful titles to enable them to search for documents by title, whereas others do not use meaningful titles and so would not desire a search feature that prioritised presence in the title. A common theme that came out was that the participants would be looking to search specifically within their own work rather than broadly. They placed value on being able to either find specific pieces of work based on a chemical structure or a piece of equipment, or being able to pull together all the different documents (including different formats of documents) for the same project or experiment. A lot of advanced search features were requested, mainly around searching for the types of tags (e.g. dates, experiment types, experiment numbers, units), so a tagging system where the tags themselves had types and searching could be done on tag type would be both useful and easy to implement and expand if the search was written in such a way that it searched on tag term and type.

A common theme across both tagging and searching was that participants were looking to use them to enhance their performance by essentially providing a faster way of searching through and collating together their material for continued experiments and write-ups. The participants were only in favour of using a tagging system if it provided these features, and was simple and efficient to set up. Unfortunately the participants were also aware that the current tagging systems demonstrated in Semanti-Cat were not sufficient for their needs, and that they anticipated needing to edit the tags and tooltip descriptions if they wanted to build up a useful set of document tags and improve their search capabilities; thus creating a circular problem. Any tagging software therefore needs to be able to tag well enough that the users are not initially put off by it and have enough customisation options that they can personalise it to how they want.

During the focus groups, a commonly recurring point made was some of the participants felt that the tagging and searching would be of more use to them if they were searching through somebody else’s work rather than their own. Previous research conducted by Chen et al. [50] details that novice users in hypermedia learning systems use an undirected search of trial and error, whereas an expert user will perform a directed search. The comments from participants resonated with this finding, as they described that they felt more knowledgeable about their own documents and knew how to specifically search through them, but that this behaviour would not hold true for searching the work of others. Participants noted that they would be more likely to add in extra tags or provide more detailed descriptions for the tooltips if they were handing over their work to someone else or sharing it in a group rather than doing it purely for their own personal use. These points were made on the basis that the Ph.D. students felt that they were specialised in their subject and well versed in what they were writing about, and in some cases already felt that they knew how to search through their own work.

This different attitude for shared work versus personal work illustrates that the participants understand the value of knowledge sharing and leaving their legacy; they could see the use for this type of system for work that was being passed on. Additionally, the participants pointed out that some of them felt that this type of system would be very useful for undergraduates who did not have as much knowledge as they did and who also worked on a wider variety of subjects. This range of subjects would elicit different tags, rather than postgraduate work, which can be specialised to one or two main subject areas. Furthermore, the Ph.D. students explained that at their level of experience they did not necessarily require common terms to be explained, but that they would have appreciated it when they had less experience in their subjects. Both of these themes can be looked at under the guise of ‘novice’ and ‘expert’ users, in that even a Ph.D. student who is an expert in their subject area could still be a novice in another subject area, and return to having a similar level of knowledge in that field as an undergraduate would in their fields. Therefore it is important to consider an advanced directed search for ‘expert’ users, and an intuitive search that allows ‘novice’ users to perform an undirected search through unfamiliar work.

With regards to semantic web technologies improving the likelihood of digitisation, the comments made by the participants demonstrated a genuine need for these technologies, but with respect to improving their knowledge management capabilities, rather than a technology that would sway them to drastically increase the amount they digitised their work.

## Key findings

This section summarises the key findings based on the results and discussion.

### KF1: ELNs are still primarily perceived as a replacement for, rather than a supplement to the paper lab notebook

Whilst the participants gave some positive responses to understanding the benefits of incorporating semantic web technologies into an ELN, they still remained against the idea of giving up their paper lab notebook, irrespective of the alternative. One participant specifically commented that there was nothing that could be done to sway them to an ELN. However, comments were also made that noted that participants would use this software to add to what they already had, if it didn’t require giving up their paper lab notebook. Additionally, when the participants were describing how they might use this type of software phrases like ‘knowledge management tool’, ‘knowledgebase’ and ‘project management tool’ were mentioned, highlighting that the participants didn’t see this type of software as an ELNs so much as an additional organisational tool that could help them with their currently digitised work. How the participants view this type of software is vitally important as perhaps a simple way of encouraging more scientists to use it would be to market it under a slightly different bracket of software. Furthermore, when disassociating the notion of replacing the paper lab notebook, and looking at the software as just another tool they could use, their responses about using it became more positive, and some clear software based needs were identified.

### KF2: Tagging and searching a scientist’s work is also a personal endeavour

The participants made it very clear that they all would tag and search their work in different ways, agreeing with the findings from previous research [4, 7, 26, 51]. Some common themes were requested with regards to tags, but even in that instance different participants prioritised different types of tags more. Similarly, when asked which criteria the participants would expect to be prioritised with regards to searching, the participants varied in their answers due to the nature of how they organise their work (e.g. some do not use meaningful titles so wouldn’t expect presence in the title to be prioritised in a search, but others would because they title their work in a way that is meaningful to them), and various different types of advanced search features were requested. This suggests that a one size fits all approach to this problem wouldn’t work. Instead the tagging and searching would need to be designed in such a way that it was customisable, such that users would have a lot of control over what they opted to have tagged in the first place. The level of customisation required suggests that machine learning could be of use here, if the tags and searches were fed into a training set for the software to learn how their users wanted their work to be tagged and how they would use the search bar.

### KF3: Some scientists attribute tagging and enhanced searching as a more useful feature when searching through other scientists work rather than their own

When the types of tags were discussed the participants said that they would potentially add detailed tag descriptions or add extra tags if they were handing over their work to someone else or sharing it in a group rather than doing it for themselves. These points were made on the basis that the Ph.D. students felt that they were specialised in their subject and well versed in what they were writing about, and in some cases already felt like they knew how to search through their own work. It also shows however that they understand the value of knowledge sharing and leaving their legacy and that they could see the use for this type of system for work that was being passed on. Additionally, the participants pointed out that some of them felt like this type of system would be very useful for undergraduates who had less specialised knowledge.

### KF4: Irrespective of whether they want to use an ELN, scientists require improved knowledge management systems

Not all scientists currently want to use an ELN, but that doesn’t meant that they don’t want software to improve their knowledge management. Comments were made that denoted that a participant would not be swayed to an ELN but would use software with these technologies to add to what they already had. When the participants were describing how they might use semantic web tools, phrases like ‘knowledge management tool’, ‘knowledgebase’ and ‘project management tool’ were mentioned, highlighting that the participants saw a genuine value in having an additional organisational tool that could help them with their currently digitised work.

## Conclusions and future work

This section begins with the overarching conclusions that have been drawn from the work conducted in this paper. Following this, the conclusions derived for each research questions will be answered.

### Overarching conclusions

The overall feedback from the focus groups were that the participants typically liked the idea of tagging and searching as long as the material was accurate enough to aid their work efficiency, and was easy to use. Participants understood that there was a merit in being able to organise and manage all of their work in once place and easily search for all of the material on one experiment or project, and being able to search very specifically throughout their work would be very useful. It was also highlighted that the tagging and markup descriptions could prove very useful for collaborative work or for processing other scientists’ work to immediately gain more information on it, thus improving how scientists work with each other and transfer knowledge.

### RQ1: What are the potential advantages of using semantic web technologies to manage scientific research?

The results of this study have demonstrated that semantic web technologies can provide links between related documents and facilitate a more advanced search. Whilst the tagging that was trialled in the proof of concept system was met with mixed reactions, the participants expressed interest in the idea of tagging their work and making it more searchable, they just wanted more refined tags. They made it clear that they saw a use in being able to organise and manage all of their work in once place and easily search for all of the material on one experiment or project, or being able to search very specifically throughout their work would be very useful. With further work on the tagging and searching, potentially incorporating some machine learning techniques to train a system to learn how the different users tag and search semantic web technologies could be used effectively to aid with the organisation and management of scientists’ records. Additionally, a lot of the scientists said that they felt that semantically tagging work and adding descriptions and mark-up would be very useful for collaborative work or for processing other scientists work to immediately gain more information about it, therefore semantic web technologies could also be used to improve how scientists work with each other and transfer knowledge.

The participants also highlighted some further use cases of using Semantic Web Technologies to manage their scientific research. It was suggested that using semantic annotations that gave descriptions of the terms would be useful as an undergraduate teaching tool, as it would be useful for less experienced scientists to be able to see meanings of common scientific terms in their documents. Producing links between documents that used the same compounds was also highlighted as a use case, as was being able to identify the new chemicals that a scientist had made during their experiments, so that these could be easily searched on.

### RQ2: What tools and technologies are the most effective for using semantic web technologies to manage scientific research?

The results of the user studies demonstrated that the existing tools for semantic annotation were not sufficient; some tools were more useful than others but ultimately would require significant improvement to prove useful. With respect to automatic chemical recognition, GATE was not well received with regards to its ability to recognise chemicals. GATE tags only compound formulas (e.g. SO2, H2O, H2SO4 ...), ions (e.g. Fe3+, Cl-) and element names and symbols (e.g. Sodium and Na). Limited support for compound names is also provided (e.g. sulphur dioxide) but only when followed by a compound formula (in parentheses or commas) [52]. ChemicalTagger was better received, as it tags both chemical elements and compounds, and recognises many more chemicals than GATE, however it would still require some significant enhancements (potentially using natural language processing) to refine how chemicals are recognised. The participants also noted that whilst ChemicalTagger picked out chemicals better, it picked out too many of them that wouldn’t be useful as they were common across all of their documents. To improve the effectiveness of this tool, further processing should be done to store the regularly used chemicals as metadata and keep any rarely used chemicals across the document corpus or new chemicals as tags.

OpenCalais had the potential to be useful, but it was clear that it was too broad and didnt give enough domain specific terminology. Similarly, the participants found that aligning their document terms with ontology terms picked out some useful tags, but again was too broad, and the use of ontologies that weren’t very specialised to their domain was not ideal. It was also clear from the requirements of the users that other types of tagging services would need to be incorporated so that other types of terms (e.g. dates) and different types of documents could be recognised. These techologies would have a greater change of being useful if additional ontologies were identified and added; and OpenCalais was enhanced with some additional natural language processing to identify the main discipline, with which only certain ontologies would be used. Similarly, further natural language processing could be done to narrow down the tags extracted by OpenCalais tags.

### RQ3: What are the needs and requirements of scientists with regards to knowledge management of their research?

Whilst semantic tagging and subsequent semantic searching is highly desired by scientists, the notion of simply ‘tagging’ is anything but simple. The tagging process and how scientists wish to organise and search through their work is highly personalised, and a common factor across all participants in the user studies was that a ‘less is more’ approach is needed; with scientists requiring a small amount of highly customised tags that are specific to their discipline.

Similarly there was an obvious need to differentiate between tags/classifications and metadata. The participants demonstrated that whilst there could be uses to storing metadata about all the chemicals in a document (e.g. if there was an issue with a specific chemical, all the experiments that used that chemical could be quickly and easily searched for), these related terms shouldn’t all be stored as tags. Some additional natural language processing would need to be performed over the document to not only pick out key terms, but to then ascertain which are unique enough to be used as tags.

This demonstrates that a one-size-fits-all approach to this problem would not work. Instead the tagging and searching would need to be designed in such a way that it was customisable, such that users would have a lot of control over what they opted to have tagged in the first place. The level of customisation required suggests that machine learning could be of use here, if the tags and searches were fed into a training set for the software to learn how their users wanted their work to be tagged and how they would use the search. As Participant S put it, you don’t search your own work broadly! Typically scientists will search through their own work very specifically, and therefore the use of semantic web technologies to facilitate tagging and searching needs to enable that. Finally, using Ph.D. students as participants also highlighted how disparate different scientists work can be, even working in the same group. Therefore it is vital that specific domain knowledge is used in these processes (e.g. the ontologies that align strongly with their work) if these technologies are going to prove useful for knowledge management.

### RQ4: Would the use of semantic web technologies convince scientists to digitise their research further?

The main conclusion from this body of work is that using semantic web technologies in the ways described in this paper would have a much greater influence on improving the management of the scientific record rather than vastly furthering the digitisation of it (by encouraging further ELN Usage). The study participants said that they saw this type of software as more of a knowledge management or organisational tool, and expressed a desire for a tool such as this as long as it actually provided useful functionality. However, the participants mostly agreed that this tool wouldn’t necessarily entice them to further digitise their work, as some of the participants were against the idea of digitising their work more than they already do, and others said that they thought their work was already digitised to an appropriate state, or as much as it could be.

Furthermore, scientists are still attached to their paper lab notebook. The user studies undertaken in this paper and its precursor [7] illustrated that there were many adoption barriers to adopting an ELN that still hold true, including the disruption it would have to current working practices, and the hostile lab environment towards technology. Therefore, this clearly isn’t just a software issue and software in itself could not mitigate these barriers. However, a significant need that was elicited from multiple user studies was that a lot of the scientists’ work (even all of their digital work) isn’t cohesively organised, and is stored in multiple different formats and locations using different software and as of yet there isn’t an overarchingplatform that allows them to collate all of these records together in a useful manner. Adding semantic tags of different types to tag and categorise different documents would enable scientists to link their documents to their data and to easily find material related to one experiment to facilitate an easier write-up.

For a majority of the participants in the focus groups it seemed like the management element was where they saw a benefit to this type of software. Many participants intimated or directly said that this wouldn’t encourage them to further digitise their work, even though they would consider using it on their already digitised work. These participants were all but one of the biologists, one of the physicists and half of the chemists. However, others were more in favour of digitising their work and had actively been taking steps to do so. Interestingly the participants who showed a higher proclivity towards digitising their work were two physicists who were nearing the end of their Ph.D.s and had started working in industrial labs, and a chemist and biologist both of whom had supervisors who were in favour of ELNs (although the chemist was nearing the end of their Ph.D. and the biologist was a first year Ph.D. student). The rest of the chemists sat in the middle of these opinions, and weren’t against the idea of further digitising their work but were more sceptical that any system could provide them with the requirements they would actually want. The discussions in the focus groups suggested that something more than just a new piece of software would be required to either consider removing paper from the lab or to drastically increase the amount that scientists digitised their work, such as starting these types of practices earlier and for supervisors and employers to set them on this road as early as possible to ensure that they get into good habits from the start.

## Future work

It is clear that further research needs to be conducted around using semantic web technologies in a substantially more personalised manner. Further research and work should be conducted on semantic tagging and searching capabilities, potentially incorporating some machine learning techniques to train a system to learn how the different users tag and search. Research should also be done into the best ways to classify types of documents and more generic tagging/text processing services should be identified to pick out less domain specific terms such as dates. A future prototype could be created to provide a discussion aid for a new set of focus groups to iteratively evaluate whether these approaches would be better met by scientists. This time a more extensive user study should be conducted; ideally several studies would be conducted for participants to extensively test out the different features of the software, both domain and semantic, to see how the domain based add-ons fared with regard to actually writing up their work, and how well the semantic layer tagged them. Following that some focus group discussions should also be conducted to evaluate, in groups, how well the implemented functionality meets their requirements.

Additionally, this paper demonstrates that whilst there is a need for superior knowledge management processes, even making these improvements wouldn’t necessarily entice scientists to digitise their work further. Therefore it would be worth conducting user studies to understand how a hybrid notebook could work, where paper and technology can be used together. It would be interesting to explore different methods of increasing digitisation through unconventional methods such as taking photographs of lab pages and automatically saving them to note booking software such as Google Drive or OneNote. Simple software could be written to automatically organise lab notebook pages into dated folders, which would only require the users to take a photograph of each lab page using a phone app. This could then link to the users note booking software of choice such that whilst writing up their work they could easily access photographs of their lab books. Alternatively, Smart Paper and Pens Systems such as Bamboo 2 could be trialled. This system provides special surface for users to place their paper notebooks on, and a special pen to write on the paper and save their notes to a computer via a mobile app. This would allow users to use the affordances of paper notes whilst still allowing them to be digitised. Trialling these different methods that do not aim to replace paper, but aim to work with it to improve the digitisation of the scientific record could lead to new strategies towards ensuring that the scientific record is digitised and maintained for prosperity.

## Additional file

**Additional file 1.** A file describing how to access the focus groups questions and transcripts.

## Abbreviations

ELN: electronic lab notebook
ELNs: electronic lab notebooks

## Footnotes

1 https://discussion.evernote.com/topic/39748-context-booster-your-knowledge-assistant-for-evernote/.

2 Text/raw was chosen because Semanti-Cat used the free version of the OpenCalais API, and based on the results of the previous user studies conducted in Kanza et al. [7] with a similar pool of participants, they were most likely to produce word or PDF documents, both of which could be converted to .txt documents.

3 Given that the papers would not be news stories, and this project used the free version which did not support PDF, this was not set and was left to the default value.
